# Cross-species chromosome painting tracks the independent origin of multiple sex chromosomes in two cofamiliar Erythrinidae fishes

**DOI:** 10.1186/1471-2148-11-186

**Published:** 2011-06-30

**Authors:** Marcelo B Cioffi, Antonio Sánchez, Juan A Marchal, Nadezda Kosyakova, Thomas Liehr, Vladimir Trifonov, Luiz AC Bertollo

**Affiliations:** 1 Departamento de Genética e Evolução, Universidade Federal de São Carlos, São Carlos, Brazil; 2 Departamento de Biología Experimental, Universidad de Jaén, Jaén, Spain; 3 Institute of Human Genetics, Jena University Hospital, Jena, Germany; 4 Institute of Chemical Biology and Fundamental Medicine, Novosibirsk, Russia

**Keywords:** chromosome painting, microdissection, fish cytogenetics, sex chromosome evolution, Erythrinidae fish

## Abstract

**Background:**

The Erythrinidae fish family is characterized by a large variation with respect to diploid chromosome numbers and sex-determining systems among its species, including two multiple X_1_X_2_Y sex systems in *Hoplias malabaricus *and *Erythrinus erythrinus*. At first, the occurrence of a same sex chromosome system within a family suggests that the sex chromosomes are correlated and originated from ancestral XY chromosomes that were either homomorphic or at an early stage of differentiation. To identify the origin and evolution of these X_1_X_2_Y sex chromosomes, we performed reciprocal cross-species FISH experiments with two sex-chromosome-specific probes designed from microdissected X_1 _and Y chromosomes of *H. malabaricus *and *E. erythrinus*, respectively.

**Results:**

Our results yield valuable information regarding the origin and evolution of these sex chromosome systems. Our data indicate that these sex chromosomes evolved independently in these two closed related Erythrinidae species. Different autosomes were first converted into a poorly differentiated XY sex pair in each species, and additional chromosomal rearrangements produced both X_1_X_2_Y sex systems that are currently present.

**Conclusions:**

Our data provide new insights into the origin and evolution of sex chromosomes, which increases our knowledge about fish sex chromosome evolution.

## Background

Fluorescence in situ hybridization (FISH) using whole chromosome-specific probes (wcp) is an important cytogenetic tool to study the origin and evolution of sex chromosomes in several organisms [1-8]. The diversity of sex-determining mechanisms, as well as the absence of heteromorphic sex chromosomes in many fish species make this group a useful model to study the evolution of vertebrate sex chromosomes [9,10]. However, research involving chromosome painting in fish is rarely performed because it is difficult to obtain the necessary probes. The few available studies are focused on karyotypic [11,12] and sex chromosome evolution [2,5,7,13-15]. The current literature suggests that a variety of sex-determining mechanisms and sex chromosomes may have evolved independently in different fish species.

Erythrinidae is a Neotropical fish family that is characterized by species that have a wide variety of chromosomal forms, as well as a wide range of distinct sex chromosomes. The red wolf fish *Erythrinus erythrinus *(EER) is karyotypically diverse among different populations, with four currently identified karyomorphs (A to D) [16]. Karyomorph A is characterized by 2n = 54 chromosomes that have very similar karyotypic structures and the absence of heteromorphic sex chromosomes. Karyomorphs B, C and D share an X_1_X_1_X_2_X_2_/X_1_X_2_Y sex chromosome system, but they can differ in the diploid number and chromosomal morphology. Karyomorph D has 2n = 52 chromosomes in females and 2n = 51 in males; previously published data suggests that karyomorph D was derived from a karyomorph A-like system [17]. In fact, although there are differences in the diploid number between karyomorphs A and D and only karyomorph D has a differentiated X_1_X_1_X_2_X_2_/X_1_X_2_Y sex system, they share a relatively similar karyotypic structure that is characterized by several acrocentric chromosomes and a few bi-armed chromosomes. Additionally, mapping of distinct classes of repetitive sequences (5S rDNA, Cot-1 DNA, *Rex3 *and telomeric repeats) in the centromeric region of the Y chromosome indicated that a centric fusion between acrocentric pair number 5 and 12 in karyomorph A led to the formation of these sex chromosomes, in addition to the unpaired X_1 _and X_2 _chromosomes in the male karyotype of karyomorph D [17].

Similarly, the wolf fish *Hoplias malabaricus *(HMA) also demonstrates significant karyotypic diversity and well-defined population differences in the diploid number, chromosome morphology and sex chromosome systems. Currently, seven easily distinguishable karyomorphs (A to G) have been identified [18]. Three well-differentiated sex chromosome systems occur in this group, namely XX/XY in karyomorph B, X_1_X_1_X_2_X_2_/X_1_X_2_Y in karyomorph D and XX/XY_1_Y_2 _in karyomorph G; additionally, karyomorph C has an early differentiated XX/XY system [18,19]. Karyomorph D has 2n = 40 chromosomes in females and 2n = 39 chromosomes in males; repetitive DNA chromosomal mapping suggests that karyomorph D may have derived from a karyotype similar to karyomorph C, which is characterized by 2n = 40 chromosomes in both sexes [19,20]. A conspicuous proximal GC-rich heterochromatic/18S rDNA site, which is present on the long arms of the X and Y chromosomes in karyomorph C, is also located in the same region on the X_1 _chromosome and the short arm of the large Y chromosome of karyomorph D; this suggests that the X_1 _and Y chromosomes of karyomorph D derived from the XY chromosomes of karyomorph C. In addition, the Y chromosome of karyomorph D shares similar DNA sequences with chromosomes Y and 20 of karyomorph C. Chromosomal pair number 20 in karyomorph C and its homolog in karyomorph D (X_2_) have centromeric satellite 5S *Hind*III-DNA, as well as an exclusive interstitial site that is present on the long arms of the Y chromosome of karyomorph D, which is the only non-centromeric location of this DNA sequence in the entire karyotype. These data indicate that this interstitial site is derived from the centromere of chromosome 20, which was fused to the ancestral Y chromosome in karyomorph C and resulted in the dicentric Y chromosome currently present in karyomorph D [19]. Moreover, additional studies have shown that this dicentric Y chromosome behaves as a stable component of the karyotype having a correct segregation during meiosis [20].

In the present study, we analyzed the origin of the EER and HMA X_1_X_2_Y sex chromosomes by performing chromosome painting analysis with sex-chromosome-specific probes established by microdissection. The X_1 _chromosome of HMA (karyomorph D) and the Y chromosome of EER (karyomorph D) were microdissected and wcp-FISH was performed on the EER (karyomorphs A and D) and HMA (karyomorphs C and D) chromosomes. The results characterized the chromosomes that gave rise to the multiple sex determination systems and that both sex systems originated from different autosomal pairs. Our data provide new insights into the origin and evolution of sex chromosomes in fish, which increases our understanding of vertebrate sex chromosome evolution.

## Results

### Hm-X1 probe hybridization to HMA chromosomes

**Figure 1:**
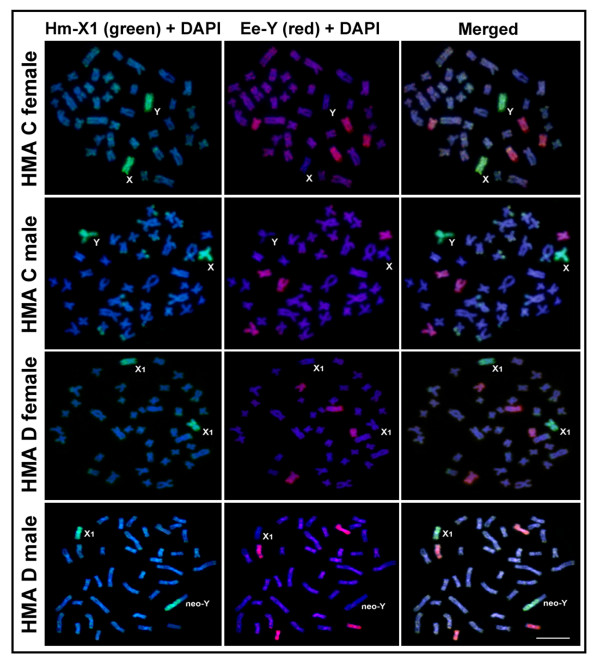
**Chromosome painting with the HMA-derived Hm-X_1 _(green) and EER-derived Ee-Y (red) probes**. The probes hybridized to the female and male metaphase chromosomes in *Hoplias malabaricus *(HMA) karyomorphs C (2n = 40, XX/XY) and D (2n = 40/39, X_1_X_1_X_2_X_2_/X_1_X_2_Y). Note that the Hm-X_1 _probe hybridized completely to the poorly differentiated X and Y chromosomes in karyomorph C, as well as the X_1 _chromosome and a large extension of the neo-Y chromosome in karyomorph D. The EER-derived Ee-Y probe entirely labeled two distinct submetacentric autosomal pairs of HMA chromosomes. Scale bar = 5 μm.

In karyomorph C, the X_1 _chromosome-specific probe (Hm-X1) hybridized to both of the X chromosomes in females and the X and Y chromosomes in males (Figure 1). When the Hm-X1 probe was hybridized to karyomorph D, two chromosomes were completely painted in females, corresponding to both X_1 _chromosomes; in males, one chromosome was completely painted, corresponding to the X_1 _chromosome, and half of another chromosome was painted, corresponding to the Y chromosome (termed neo-Y). No signal was observed on the X_2 _chromosome (Figure 1). In general, there was a uniform signal for all of the sex chromosomes except X_2_, which indicates that there is high homology between their euchromatic and heterochromatic regions. Additionally, faint hybridization signals were observed in the subtelomeric heterochromatic segments of some autosomes, which may possibly be due to shared repetitive sequences. These results suggest that the painted chromosome pair in karyomorph C is the ancestral chromosome pair for the sex chromosome system observed in karyomorph D.

### Ee-Y probe hybridization to EER chromosomes

**Figure 2:**
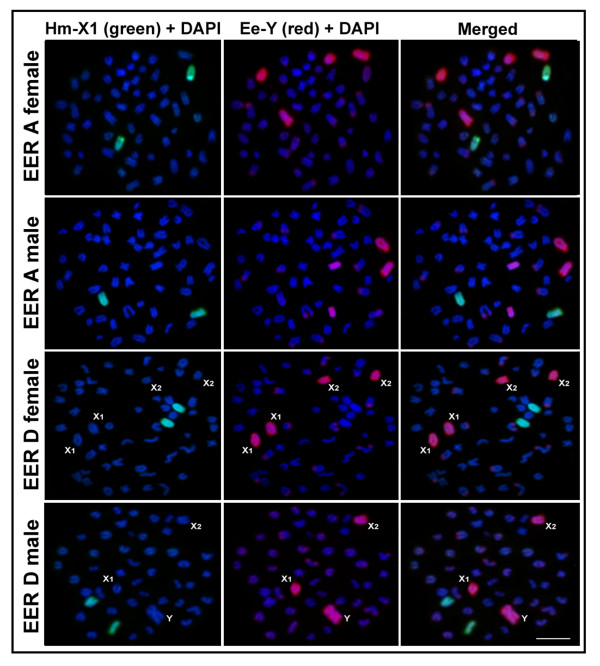
**Chromosome painting with the Hm-X1 (green) and Ee-Y (red) probes**. The probes hybridized to the female and male metaphase chromosomes in *Erythrinus erythrinus *(EER) karyomorphs A (2n = 54, both sexes) and D (2n = 52/51, X_1_X_1_X_2_X_2_/X_1_X_2_Y). Note that the Ee-Y probe hybridized to four acrocentric chromosomes in EER karyomorph A, as well as the X_1_, X_2 _and Y chromosomes in EER karyomorph D. The HMA derived Hm-X1 probe hybridized to a distinct autosomal pair in EER. Scale bar = 5 μm.

The Y-chromosome-specific probe (Ee-Y) completely painted two chromosome pairs in both males and females of karyomorph A (Figure 2), as well as the X_1 _and X_2 _chromosomes in females and the X_1_, X_2 _and Y chromosomes in males of karyomorph D (Figure 2). Weak signals were observed in the subtelomeric heterochromatic segments of some autosomes, which indicate that these chromosomes may have similar repetitive sequences. These results suggest that the painted chromosome pairs in karyomorph A are the ancestral pairs for the sex chromosome system observed in karyomorph D.

### Reciprocal cross-species FISH

None of the distinguishable sex chromosomes in either species displayed consistent hybridization signals with the cross-FISH experiments. In HMA, the Ee-Y probe hybridized completely to a medium and small autosomal submetacentric pair in males and females of karyomorphs C and D (Figure 1). In EER, the Hm-X1 probe hybridized to a medium-sized autosomal acrocentric pair in males and females of karyomorphs A and D (Figure 2). The reciprocal cross-species FISH experiments (the Hm-X1 probe with EER and the Ee-Y probe with HMA, respectively) clearly show that hybridization only occurred with autosomes in all of the karyomorphs that were analyzed.

## Discussion

### Origin of the sex chromosome systems in HMA karyomorphs

The complete staining of both the X and Y chromosomes with an HMA-derived X1 probe for karyomorph C indicates that these sex chromosomes are highly similar. These results confirm the previous hypothesis that the XY chromosomes of HMA karyomorph C are at an early stage of differentiation. Indeed, a previous study has indicated that these chromosomes differ only by a slight amplification of repetitive sequences on the X chromosome [19].

**Figure 3:**
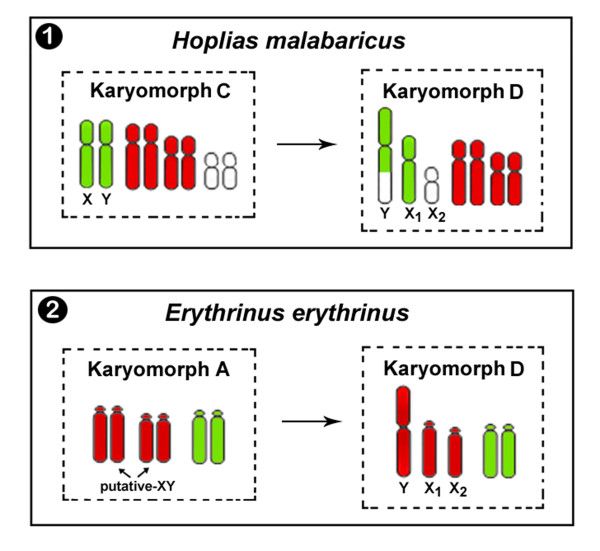
**Overview of the proposed independent evolution of the X_1_X_2_Y sex chromosomes in HMA and EER based on karyotypic features and cross-FISH results with sex chromosome wcp**. **(1) **The HMA X_1_X_2_Y system originated by tandem fusions between the poorly differentiated Y chromosome of HMA karyomorph C and an autosome, which created the large neo-Y chromosome in HMA karyomorph D. The unpainted extension of the neo-Y corresponds to the ancestral homolog of the X_2 _chromosome, which lacks homology with the X_1 _chromosome. **(2) **The same sex system in EER derived from a centric fusion between a morphologically undifferentiated Y chromosome in EER karyomorph A and an autosome, which gave rise to the large neo-Y chromosome in EER karyomorph D. Although the XY chromosomes have not been cytogenetically identified in EER karyomorph A, they are presumed to be one of the two acrocentric pairs that wholly hybridized to the Ee-Y probe. The chromosomes painted with the Hm-X1 and Ee-Y probes are indicated in green and red, respectively. Note that although the HMA and EER chromosomes share sequence homology, the two sex chromosome systems have evolved independently.

When hybridized with HMA karyomorph D, the HMA X1 probe painted both of the X_1 _chromosomes in females, as well as the entire X_1 _chromosome and half of the neo-Y chromosomes in males. However, we did not detect hybridization with the X_2 _chromosome, which indicates that the X_1 _and X_2 _chromosomes lack sequence homology and are likely unrelated chromosomes. We hypothesize that the unpainted region of the neo-Y chromosome corresponds to the X_2 _chromosome. Thus, the multiple X_1_X_2_Y system in HMA originated through a tandem fusion between the proto-Y chromosome conserved in karyomorph C and one autosome, which created the large neo-Y chromosome that is characteristic of karyomorph D and one additional unpaired chromosome that was renamed X_2 _**(see **Figure 3).

### Origin of the sex chromosome systems in EER karyomorphs

For EER karyomorph D, the EER-derived Ee-Y probe hybridized to the large metacentric Y chromosome, as well as the entire acrocentric X_1 _and X_2 _chromosomes in both male and female karyotypes. These results indicate that the Y metacentric chromosome arose from a centric fusion between two non-homologous acrocentric chromosomes, which were renamed X_1 _and X_2_. Additionally, the Ee-Y probe hybridized to four acrocentric chromosomes in males and females of EER karyomorph A. This indicates that the multiple sex system of EER karyomorph D derived from a karyomorph A-like ancestor through chromosomal rearrangements involving a putative sex pair that was morphologically undifferentiated and a pair of autosomes **(see **Figure 3). Although the sex chromosomes remain cytogenetically unidentified in karyomorph A, they are likely one of the acrocentric pairs that was entirely stained by the Ee-Y probe.

Thus, in both species, reorganizations between undifferentiated or poorly differentiated sex chromosomes and autosomes gave rise to the multiple sex chromosome systems. Rearrangements between sex chromosomes and autosomal pairs have previously been reported in several organisms [3,16,17,21-24].

Chromosomal rearrangements during the evolution of multiple sex chromosomes systems may reduce the need for alternative mechanisms to suppress recombination at breakpoint sites [25]. In the systems described here, the tandem and centric fusions were crucial steps for the origin of the X_1_X_2_Y sex systems in HMA and EER, respectively; no further differentiation appears to have occurred between the sex chromosomes within each species. Thus, the primary chromosomal rearrangements that occurred during the origin of these multiple sex chromosome systems appear to have been sufficient to fix the resulting chromosomes in the respective populations.

### Independent origin of X_1_X_2_Y sex systems in Erythrinidae fishes

One interesting feature of fish sex chromosome biology is that only a few species present cytogenetically differentiated sex chromosomes. Nonetheless, XY sex systems are not common in fish. Because both of the fish species analyzed here are closely related and have similar multiple sex chromosomes that were derived from incipient XY systems, we were interested in comparing the origin of both sex systems. Specifically, we were interested in whether the systems independently evolved or formed prior to the divergence of the *Hoplias *and *Erythrinus *genera. We used reciprocal cross-species FISH with X and Y probes to analyze the origin of the sex systems in these two genera. The absence of a signal on the recognizable sex chromosomes after inter-specific painting clearly indicates that the X_1_X_2_Y chromosomes evolved independently in each species and are not related. Therefore, distinct ancestral XY chromosomes and autosomal pairs were involved in the formation of the multiple sex chromosomes in each species (Figure 3).

Numerous heteromorphic sex chromosomes have evolved independently in various plant and animal lineages [26,27]. Moreover, comparative syntenic analysis supports the hypothesis that sex chromosomes have independently evolved in different vertebrate lineages [28]. In fish, a previous study that used linkage analysis with eight isolated sex-linked markers indicated that the sex chromosomes of *Oryzias javanicus *are not homologous to those of any other *Oryzias *species [29-31]. Reciprocal cross-FISH experiments using sex chromosome probes within the genus *Eigenmannia *clearly indicated that there is no homology between the X_1_X_2_Y and XY systems, which suggests that these sex chromosomes independently evolved [7]. Similar results have also been reported for the salmonoid fish, where two paint probes specific to the short and long arms of the Y chromosome of *Salvelinus namaycush *hybridized to two different autosomal chromosome pairs in *Oncorhynchus mykiss *and *O. tshawytscha*; again, these data indicate that there is no homology between the sex chromosomes of these two closely related genera [2]. These findings suggest that a variety of sex-determining mechanisms and sex chromosomes have independently evolved several times in the various fish genera. The most plausible explanation for the independent origin of sex chromosomes in fish is that different primary sex-determining genes may have evolved on different sex chromosomes, where an autosomal gene or a duplicated gene copy acquired a new mutation and gave rise to either male or female development; this would result in a novel sex-determining gene and produce new sex chromosomes from different autosomes [26].

## Conclusions

In summary, we analyzed X_1_X_2_Y sex chromosome evolution in HMA and EER fish species. Our data indicate that there is a high plasticity of sex determination mechanisms in fish. It is noteworthy that two cofamiliar species with the same type of multiple sex chromosome system that emerged from poorly differentiated XY sex chromosomes have independently evolved and have gone through distinct differentiation processes. This data highlight the potential role of studies conducted in fish models to better understand the process of vertebrate sex chromosome evolution.

## Methods

### Specimens and chromosome preparation

In this study, we analyzed HMA samples of karyomorphs C and D, and EER samples of karyomorphs A and D. We studied a total of 85 specimens (38 females and 47 males). Overall, 8 HMA karyomorph C females and 10 males were collected from the Bento Gomes River (Poconé, Mato Grosso State, Brazil); 11 karyomorph D females and 12 males were collected from the Monjolinho stream (São Carlos, São Paulo State, Brazil). Additionally, 9 EER karyomorph A females and 12 males were collected from the Tietê River (Penápolis, São Paulo State, Brazil), and 10 EER karyomorph D females and 13 males were collected from the Pirangi River (Parnamirim, Rio Grande do Norte State, Brazil). The specimens were deposited in the fish collection of the Cytogenetic Laboratory, Departamento de Genética e Evolução, Universidade Federal de São Carlos, Brazil. Mitotic chromosomes were obtained from cell suspensions of the anterior kidney using the conventional air-drying method [32]. Approximately 30 cells were analyzed per karyomorph to assess the diploid number. The experiments followed ethical protocols, and anesthesia was administered prior to sacrificing the animals.

### Chromosome microdissection

**Figure 4:**
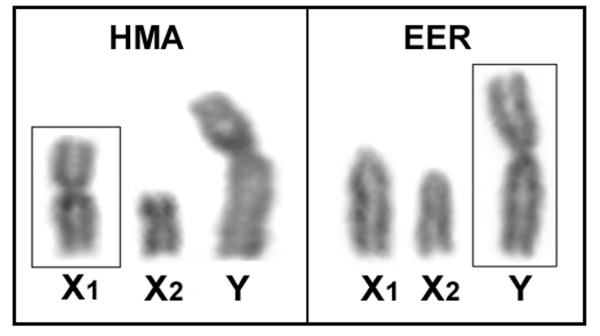
**Multiple X_1_X_2_Y sex chromosomes of *Hoplias malabaricus *(HMA) and *Erythrinus erythrinus *(EER)**. The X_1 _chromosome of HMA and the Y chromosome of EER that were microdissected and used for probe construction for wcp-FISH analysis are boxed.

Eighteen copies of the X_1 _chromosome and fifteen copies of the Y chromosome were microdissected from male HMA karyomorph D and EER karyomorph D metaphase plates, respectively (Figure 4), using the methodology previously described with minor modifications [33]. In contrast to the large metacentric Y chromosome of EER, which is easily identifiable after Giemsa staining, the HMA chromosomes were stained with Chromomycin A_3 _to allow for precise identification of the X_1 _chromosomes by identification of a differential GC-rich heterochromatic block adjacent to the centromeric region on the long arm.

Briefly, the chromosome suspensions were dropped onto pre-cleaned coverslips and subjected to regular Giemsa or Chromomycin A_3 _staining. The microdissection was performed using an inverted microscope (Zeiss Axiovert 135) with a sterile glass needle attached to a mechanical micromanipulator (Zeiss). The glass needles were prepared with a pipette puller model PB-7 (Narishige, Japan), and the chromosomes were transferred to a micropipette containing a collection solution (10 mM Tris-HCl pH 7.5, 10 mM NaCl, 0.1% SDS, 1 mM EDTA pH 7.5-8.0, 0.1% Triton X-100, 1.44 mg/ml proteinase K and 30% glycerol). The solution was subsequently transferred to a 0.5 ml Eppendorf tube containing 0.63 μl of Sequenase buffer (USB), 0.4 μl of 0.2 mM dNTPs, 0.6 μl of 40 mM DOP primer (5'-CCGACTCGAGNNNNNNATGTGG-3') and 3.37 μl of PCR water per sample.

The first eight cycles of DOP-PCR were conducted using sequenase T7 DNA polymerase (USB, Cleveland, USA) under the following program: 90°C for 1 min, 25°C for 2 min and 34°C for 2 min. An initial denaturation step at 92°C for 5 minutes was included to inactivate the proteinase K. A sequenase mix (0.2 μl) containing 12 Uμl^-1 ^T7 DNA polymerase and 1.75 μl of Sequenase dilution buffer was added at each cycle during annealing. The reaction volume was increased to 50 μl by adding 0.1 U Taq polymerase, 0.2 mM dNTPs, 20 μM DOP primer, 25 mM MgCl_2 _and 34.23 μl of PCR water. Subsequently, 33 additional cycles were conducted with the following program: 92°C for 1 min, 56°C for 2 min, 72°C for 2 min and a final 5 min extension step at 72°C.

We refer to these two probes as Hm-X1 (for the HMA X_1 _probe) and Ee-Y (for the EER Y probe).

### Fluorescence in situ hybridization

The Hm-X1 probe was PCR labeled with biotin-dUTP (Roche) and the Ee-Y probe was labeled with Spectrum-Orange dUTP (Vysis, Downers Grove, USA) with a 30-cycle label-PCR with DOP primer using 1 μl of the primary DOP-PCR products as template DNA. The FISH analyses were performed on metaphase chromosome spreads from HMA (karyomorphs C and D) and EER (karyomorphs A and D). The slides were prepared and pre-treated as previously described [33] and denatured in 70% formamide with 2 × SSC for 3 min at 72°C. For each slide, 12 μl of hybridization solution (containing 0.2 μg of each labeled probe, 50% formamide, 2 × SSC, 10% dextran sulfate and 5 μg of salmon sperm DNA) was denatured for 10 minutes at 75°C and allowed to pre-hybridize for 1 h at 37°C. The samples were allowed to hybridize for 16 h at 37°C in a moist chamber. Post-hybridization, the samples were washed with 0.4 × SSC/0.3% Igepal detergent (SIGMA) for 5 min at 73°C and 2 × SSC/0.1% Igepal for 30 s at room temperature. The biotinylated Hm-X1 probe was detected with avidin-FITC (Vector Labs, USA). The slides were counterstained with DAPI and mounted in an anti-fade solution (Vectashield from Vector laboratories). Twenty metaphase plates per karyomorph were photographed with a digital CCD camera (Olympus DP70) coupled to a fluorescence microscope (Olympus BX51).

## List of abbreviations

**2n: **diploid number; **DAPI: **4',6-diamidino-2-phenylindole; **dNTP: **deoxyribonucleotide triphosphate; **DOP-PCR: **degenerate oligonucleotide primed PCR; **dUTP: **deoxyuridine triphosphate; **EER: ***Erythrinus erythrinus; ***Ee-Y: **Y chromosome-specific probe; **FISH: **fluorescence in-situ hybridization; **HMA: ***Hoplias malabaricus; ***Hm-X1: **X_1 _chromosome-specific probe; **SSC: **Sodium Chloride-Sodium Citrate buffer; **PBS: **Phosphate-buffered saline; **WCP: **whole chromosome probe.

## Competing interests

The authors declare that they have no competing interests.

## Authors' contributions

MBC performed the experiments and drafted the manuscript. AS and JAM participated of the techniques development and contributed to the discussion of data. NK realized the chromosome microdissection and contributed to the discussion of data. TL and VT helped interpret the results and reviewed the manuscript. LACB coordinated the study, helped interpret the results and revised the manuscript. All authors read and approved the final manuscript.
